# Some Aspects of Intestinal Obstruction

**Published:** 1908-06-20

**Authors:** 


### Surgery.

### SOME ASPECTS OF INTESTINAL OBSTRUCTION.

### III.—OBSTRUCTION DUE TO CONDITIONS IN THE LUMEN OF THE BOWEL.

The passage of gall-stones must be included among the causes of chronic intestinal obstruction, although the condition so caused is usually intermediate between the acute and chronic forms. The symptoms are more acute in onset than in other forms of chronic intestinal obstruction, but are less intense than the general symptoms which attend acute strangulation of the bowel.

Biliary calculi which pass through the common bile duct are in the great majority of cases so small that they are evacuated without causing any symptoms; in rare instances they may be lodged in diverticula of the intestine and become so enlarged by the gradual deposit of phosphates as to give rise to symptoms of progressive stenosis. Sir Frederick Treves reported a case, which has now become historic, of a calculus whose nucleus was a small gall-stone. It had become enlarged by the depositing on its surface of carbonate of magnesia, which had been taken daily for medicinal purposes. Its longest diameter was one inch and a half, and it eventually gave rise to symptoms of acute intestinal obstruction. The larger calculi which lead to a more or less sudden intestinal obstruction nearly always make their way into the gut through a fistulous communication between the gall-bladder and the small intestine. By contrast, it is an almost invariable rule that when a carcinomatous gall-bladder communicates with the intestine it does so with some portion of the colon, generally, of course, in the neighbourhood of the hepatic flexure. Calculi which enter the gut in this fashion may become impacted at any point in the small intestine, or at the ileo-cæcal valve—rarely, if ever, in the colon itself. Besides this, they will tend to act in a ball-valve manner in the presence of any pre-existing stenosis, and in this way sometimes determine the acute termination of a chronic obstruction.

If the stone is just large enough to cause trouble, but not of sufficient size to become definitely impacted at any given point, the symptoms may be mild and transitory; but in other cases the complete picture of occlusion will be presented, as indicated by absolute constipation, violent pain, vomiting which tends to become stercoraceous early, and collapse. The pain is usually paroxysmal, but peristalsis of the intestine is not often seen on account of the comparatively short course of the disease. The calculus can hardly ever be palpated. A history of past jaundice with pain in the liver region and attacks of colic will strengthen the diagnosis.

Sometimes the condition undergoes a spontaneous cure, as, for instance, by the passage of a stone, which has been impacted at the ileo-cæcal valve, into the cæcum, with immediate relief to the patient; but more often the symptoms persist and remain unchanged until death occurs, if no operation is performed. This generally happens between the fifth and tenth day after the occlusion has come on.

Enteroliths, on the other hand, more often produce a genuine chronic obstruction. This is easily understood, since the enterolith is formed in the bowel by the gradual deposition of material round an originally small nucleus, and the symptoms caused by it progress slowly in severity as the size of the calculus advances. As opposed to gall-stones, they are most often found in the large intestine, where they develop in the claustra of the colon. The symptoms, which may go on for years, are indefinite and diverse, such as anorexia, digestive disturbances, constipation, occasionally very obstinate, and abdominal pain; and these are succeeded in some cases by the typical signs of a chronic obstruction.

Chronic intussusception is a cause of subacute intestinal obstruction, and the interest of these cases is as great as is their rarity. The very name of the condition is evidence of the difficulty in making a diagnosis, since, nowadays, one does not hesitate to operate upon an intussusception as soon as one is assured of its presence. In the acute variety, as seen in infants, the clinical picture presented is typical and the diagnosis is, comparatively speaking, easy. Not so, however, in the chronic form. This is somewhat vaguely defined as an intussusception in which the symptoms have existed for more than a month. It is more common in adults, as opposed to the acute form which is almost exclusively confined to children, but in both the commonest anatomical variety is the ileo-cæcal—*i.e.*, the apex of the advancing intussusceptum is formed by the ileo-cæcal valve, and it progresses at the expense of the intussuscipiens.

The clinical picture presented is very ambiguous, and the disease may, according to all accounts, drag out its course for years. The onset may be gradual, or the condition may start acutely and become chronic later. There are paroxysmal attacks of colicky pains, as in other forms of chronic intestinal obstruction, but vomiting is not a constant feature, and is usually absent until the end is at hand. There is sometimes constipation, but more often a tendency to diarrhœa with tenesmus and the passage of blood. The coils above the intussusception are hypertrophied, and visible peristalsis is generally present. A tumour whose shape is compared in most text-books of surgery to that of a sausage, so that the description has now become classical, can be felt in about 30 per cent. of cases. There is one sign on which too much reliance should not be placed. This is Dance's sign. One is said to be able to find an absence of the normal resistance in the neighbourhood of the cæcum, owing to the fact that the ileo-cæcal valve and cæcum have been shifted from their normal position. It applies, of course, only to the ileo-cæcal variety, but even in this case it is not a constant phenomenon. The patient may die from one of two conditions: he may succumb, emaciated and worn out by the frequent pain and vomiting, and the gross interference with the functions of the intestine, or he may die of an acute attack supervening on the chronic.

